# To Name or Not to Name: Eye Movements and Semantic Processing in RAN and Reading

**DOI:** 10.3390/brainsci11070866

**Published:** 2021-06-29

**Authors:** Luan Tuyen Chau, Mila Dimitrova Vulchanova, Joel B. Talcott

**Affiliations:** 1 Department of Management, University of Antwerp, 2000 Antwerp, Belgium; luan.chau@uantwerpen.be; 2 Language Acquisition and Language Processing Lab, Norwegian University of Science and Technology, 7491 Trondheim, Norway; 3 Aston Institute of Health and Neurodevelopment, Aston University, Birmingham B4 7ET, UK; j.b.talcott@aston.ac.uk

**Keywords:** semantic processing, eye movements, rapid automatized naming, dyslexia, reading

## Abstract

This study examined the well-established relationship between rapid naming and reading. Rapid automatized naming (RAN) has long been demonstrated as a strong predictor of reading abilities. Despite extensive research spanning over 4 decades, the underlying mechanisms of these causes remain a subject of inquiry. The current study investigated the role of eye movements and semantic processing in defining the RAN-reading relationship. The participants in this study were 42 English-speaking undergraduate students at a British university. The materials included a word reading task, two conventional RAN tasks (object and digit), and two RAN-like categorization tasks (object and digit). The results obtained suggested the interdependence between rapid naming and semantic processing. Hierarchical multiple regression analyses revealed that oculomotor control remains an integral part of variability in RAN and reading performance. Taken together, our results suggest that RAN and reading measures are correlated because both require rapid and accurate retrieval of phonological representations, semantic properties of visual stimuli, and stable co-ordination of eye movements.

## 1. Introduction

Rapid Automatized Naming (RAN) has long been demonstrated as a strong predictor of both concurrent and future reading abilities. In a standard RAN task, participants are shown a grid of visual stimuli that represent common objects, colors, alphanumeric, or numeric symbols and are asked to name (usually aloud) each item in the grid in a sequential order as quickly as possible. Empirical studies have shown that there is a strong correlation between the speed at which participants are able to accurately name all the items in a stimulus grid and their reading ability [1,2,3,4,5,6,7,8]. RAN is considered one of the most reliable reading assessment tools, and is widely used in literacy research because of its ease of application in different settings. It predicts literacy abilities, both in typical readers and those with reading difficulties. Moderate correlations (*r* = 0.55) have been reported between typical preschool performance on RAN and second grade word decoding [2]. Additionally, 60% to 75% of individuals struggling with reading have been suggested to exhibit RAN deficits [9,10,11,12]. The predictive power of RAN appears to persist until adulthood. Some studies have reported moderate correlations (*r* = 0.53) between performance on RAN and reading for adults aging from 36 to 65 years [13]. Significantly, this correlation between RAN and reading remains strong across different languages. RAN has been shown to be predictive of reading, regardless of their orthographic depth, and for both alphabetic and non-alphabetic languages [7,14,15,16]. 

Despite the statistical correlation between RAN and reading, the underlying mechanisms for this putative relationship remain underexplored. One approach to investigating the RAN-reading relationship is to identify the individual cognitive and linguistic components that are shared by both reading and rapid naming tasks. One of the earliest and most well-known hypotheses is that rapid naming tasks require accurate and rapid retrieval of phonological units from the mental lexicon [2]. Under this view, RAN is seen as another construct of phonological processing, meaning that efficiency in rapid naming performance is an indicator of rapid and accurate access to familiar lexical items and their phonological representations. More recent studies have indicated that rapid naming tasks may actually engage other non-phonological mechanisms and more general cognitive processes, such as attention, visual detection and integration [17]. According to Wolf & Bowers (1999), there are at least seven different sub-components that may contribute to individual variability in performance on RAN tasks: (a) attentional selection and allocation to the stimulus, (b) bi-hemispheric visual processing engaged through feature detection, visual discrimination, and pattern identification, (c) integration of visual features and pattern information with stored orthographical representations in memory, (d) integration of visual and orthographic information with stored phonological representations, (e) access and retrieval of the phonological labels for stimuli, (f) activation and integration of semantic and conceptual information with all other information, and (g) motoric responses leading to articulation [8].

Despite extensive investigation of these sub-components of RAN tasks, there remains a lack of consensus regarding which mechanisms underline the RAN-reading relationship. Norton & Wolf attempted to resolve the ongoing debate by conceptualizing RAN as a “microcosm or mini-circuit of the later developing reading circuitry” [7] (p. 429). By this theoretical account, RAN is predictive of reading because it involves a conglomeration of a common set of linguistic and perceptual processes that are also engaged whilst reading, such as phonological, orthographic, and semantic representations, integrating visual information, and allocating working memory. Performance on RAN reflects the ability to co-ordinate these interfaced processes fluently and accurately. Such a view does not raise controversy on either theoretical or empirical grounds. However, the goal in investigating the RAN-reading relation is not only to specify the underlying components, but, more importantly, to measure their contributive weight and the extent to which each component can contribute to defining the RAN-reading relationship. 

One particular unresolved question regarding the relationship between RAN and reading is the extent to which phonological processing common to both tasks sufficiently accounts for their association, or whether RAN tasks make a distinct contribution to reading beyond that explained by common phonological skills. Some researchers argue that RAN is a test mainly of the phonological component of language. Children who have deficits in phonological awareness (PA) and processing tend to experience difficulties in rapid naming tasks [18,19]. Other studies, however, have suggested that the relationship between RAN and reading cannot be sufficiently explained on the basis of a common underlying mechanism of phonological processing. One of the most influential theories in this respect is the “Double Deficit Hypothesis”, first proposed by Bowers and Wolf [20]. According to this account, phonological awareness and RAN represent separable mechanisms of reading and impairments. Children with impairments in both phonological awareness and rapid naming tasks exhibited the most severe reading impairments compared with those with deficits in either phonological awareness or rapid naming. Subsequent studies in the literature have confirmed this [8]. In addition, Swanson et al. (2003) conducted a meta-analysis on samples from 49 independent studies and reported low-to-modest correlations between phonological awareness and rapid naming in both skilled and poor readers [21]. Importantly, a recent cross-linguistic study of five languages with different degrees of orthographic complexity has established that, while RAN was a consistent predictor of reading fluency in all orthographies, the association between PA and reading was complex and mostly interactive [22]. These results suggest that, unlike PA, RAN taps into a language-universal cognitive mechanism that is involved in reading alphabetic orthographies (independent of their complexity). Neuroimaging studies have also suggested that the biological foundations for RAN and PA are not completely overlapping [17]. Cummie et al. showed that RAN performance taps into several brain regions supplementary to those that primarily engaged in phonological processing tasks, such as in the cerebellum (motor planning), middle temporal gyrus, and anterior cingulate (motor/pre-motor, supplementary motor association) [23]. 

Rapid naming tasks likely involve a complex conglomeration of linguistic, perceptual, and cognitive processes. There has been a switch to using both language and non-language components in investigating the components of RAN and in the RAN-reading relationship. One particular consideration concerning the cognitive and perceptual aspects of RAN is the role of eye movements. This is grounded on the observation that the visual scanning and serial processing of a typical RAN grid engage similar oculomotor programming to that involved in reading texts [24]. By this perspective, RAN is predictive of reading because both also involve serial processing and the ability to co-ordinate eye movements across a written page (or a grid), in addition to other language and cognitive processing [25]. Evidence in support of this comes from two main streams of research. First, several studies have shown that the statistical relationship between RAN and reading is reduced or nearly non-existent when the items on the grid are presented in isolation (discrete RAN) instead of serially [2,19,26,27,28,29,30]. Second, research from the eye-tracking literature has found that rapid naming times are strongly linked to individual differences in eye movements during word or text reading [31]. Results from these studies have revealed that longer naming times in RAN tend to be aligned with longer fixation rates, smaller saccades, increased refixation rates, and more frequent incidence of regressive saccades [25,32,33]. Moreover, Kuperman & Van Dyke found that rapid naming times are a strong predictor of all domains of per-word eye movements recorded during sentence reading [24]. Covariance between RAN performance and the percent of fixations and regressions during text reading was also found in a study by Doyle [34]. Altogether, these results indicated that higher rates of fixations and regressions rates in rapid naming tasks are associated with increased fixation and frequent regressions in word or text reading. Eye movements in rapid naming are thus analogous in many respects to those observed during reading process.

The current study aimed to examine one specific component of RAN that has received little attention so far, namely how semantic and conceptual information is activated and integrated with other sources of information in on-line processing. To the best of our knowledge, the importance of semantic and conceptual integration and activation in RAN has not been systematically investigated in typical populations. Jones et al. investigated semantic processing deficits among dyslexic readers [35], revealing difficulties both in conventional object-naming and in object-categorization tasks. Further analyses indicated that dyslexic groups experienced comparable difficulties in tasks that require semantic processing (e.g., providing verbal responses to different types of objects), as they would do in naming aloud tasks. Jones and colleagues concluded that RAN deficits arise at least in part from difficulties in semantic processing. This result implicates semantic processing as animportant potential component underlying the relationship between RAN and reading.

In this study, we adopt the logic of Georgiou et al.: “If X is the process that is responsible for the RAN-reading relationship, then increasing or decreasing the demands of X should result in an increase or decrease in the RAN-Reading relationship” [36] (p. 219). We tracked and recorded participants’ eye movements during word reading in both conventional rapid naming tasks of objects and digits, and in categorization tasks using the same stimuli. By doing so, we were also able to reassess the extent to which eye movements account for shared variance in RAN and reading tasks, in a sample of undergraduate university students with a range of reading skills typical of this population. We addressed three research questions:

1. To what extent does the activation and integration of semantic processing contribute to defining the RAN-reading relationship?
2. Do the grids of objects and digits have equivalent predictive power for word reading performance in adults?
3. In what ways are eye movements in rapid naming tasks similar to those in the word reading task?

## 2. Materials and Methods

### 2.1. Participants

A total of 42 undergraduate psychology students at a public British university between 18–25 years of age took part in the study and received course credits for their participation (*n* = 42). The participants were identified through an online portal for participant recruitment. All participants in the sample spoke English as their first languages and had normal or corrected-to-normal vision. None of the participants had reported any history of persistent cognitive impairment, reading disability, or sensory processing difficulty. All participants completed the same task battery, comprising two RAN experiments within a controlled laboratory environment.

### 2.2. Tests and Stimuli

The study consisted of modified Test of Word Reading Efficiency (TOWRE) and two sets of serial RAN stimuli (Modified Object and Digit grids). The two RAN grids were used in each of the two experimental tasks (conventional naming tasks and categorization tasks).

#### 2.2.1. Test of Word Reading Efficiency (TOWRE)

The TOWRE test was adapted from the published test version of Torgesen et al. [37] to fit the resolution and picture format required by the Tobii eye-tracker (Tobii Pro X3-120). This digital version of TOWRE consisted of 40 words in total, with 10 words each in 4 different columns. This word stimuli comprised a mixture of low and high frequency words. Less common words with more complex phonemic structure (two or more than two syllables) were concentrated in the third and fourth column. Each column had a balanced mixture of word categories (e.g., nouns, verbs, and adjectives) (See Appendix A for details).

#### 2.2.2. Serial Object Rapid Automatized Naming

The Object RAN grid set included a total of 36 visual stimuli, with 9 objects on each row. The visual stimuli were categorized into two different groups: animate objects and inanimate objects. The RAN Object grid in this study was adapted from Bone et al. (2013) [38]. To obtain balance between the number of items in each (in)animacy, we replaced “boat” and “star” with “chicken” and “frog”. The RAN grid then had an even number of animate entities (chicken, frog, fish) and inanimate entities (pencil, key, star). The same Object RAN grid was used in both Task 1 and Task 2 (See Appendix B for details). Since we were testing semantic processing in RAN by manipulating the semantic properties of the stimuli (animate versus inanimate), we need to take the psycholinguistic semantic properties of words into consideration when designing this study (as presented in Table 1 below).

#### 2.2.3. Serial Digit Rapid Automatized Naming

The Digit-RAN grid consisted of 36 digits, with 9 digits on each row. The grid contained a mixture of even numbers (2, 4, 8) and odd numbers (3, 5, 7). The idea was to compare performance on this grid with the Object RAN grid. The same Digit-RAN grid was used in both Task 1 and Task 2 (See Appendix C for details). 

### 2.3. Design

#### 2.3.1. Task 1—Conventional Naming Aloud Task

In Task 1, the participants were required to name aloud all the visual stimuli on the grid as rapidly as possible. Their eye movements were recorded using the Tobii eye- tracker.

#### 2.3.2. Task 2—Object Categorization Task

Task 2 aimed to strip away phonological processing components related to lexical access and naming. The participants were not required to name aloud the stimuli. Instead, they scanned with their eyes through the grid, examined each stimulus, and gave simple verbal responses by saying yes/no. For the Object-RAN grid, verbal responses were ‘yes’ whenever they saw a living object and ‘no’ whenever they saw a non-living object. For the Digit-RAN, participants were instructed to verbalize ‘yes’ whenever even numbers were encountered and ‘no’ if they saw an odd number.

In Task 2, we intended to observe whether semantic processing is unique to that involved in non-alphanumeric RANs only. In both tasks, we obtained measures relating to task fluency. We did not record the accuracy of participant responses, and therefore eye movement data included trials of both correct responses and errors. However, observations of participants suggested that errors of naming accuracy were rarely made by individual participants in this study of highly educated adults without a history of reading or language difficulties.

### 2.4. Procedure

Eye-tracking data were collected using the Tobii Pro X3-120 binocular eye tracker with a sampling rate of 120 Hz and analyzed using proprietary software (Tobii Studio Version 3.4.8). Participants were seated in front of a 24-inch widescreen monitor, at a distance of 50 cm from a computer screen. At the beginning of each phase of the experiment, a calibration routine was used to ensure the reliability of the eye-tracking in detecting gaze variables in relation to the computer screen upon which the stimuli were presented. Participants were also instructed to keep their heads as still as possible during the recording epochs.

The protocol for the study was approved by the Aston University Ethics Committee (#1567). Following a formal information and consent procedure, participants completed the TOWRE. This was followed by the RAN tasks. In Task 1, the participants named aloud the visual stimuli on the RAN grids, completing the Object grid first and then the Digit grid. In Task 2, the participants provided simple verbal responses to the stimuli but did not name them. As control over potential order effects, half of the participants completed Task 1 first; the other half completed tasks in the opposite order. The entire experiment took approximately 30 min to complete.

### 2.5. Variables in the Study

Seven variables were extracted from each participant dataset for each task: (a) Total Naming Time, (b) Total Fixation Duration, (c) Time engaged in Saccades, (d) Total Fixation Duration for Animate Objects, (e) Total Fixation Duration for Inanimate Objects, (f) Total Fixation Duration for Odd Digits, and (g) Total Fixation Duration for Even Digits. All timing variables are reported in seconds. Table 2 summarizes the variables in the study and indicates how we operationalize those variables in an eye-tracking study. 

## 3. Results

The unit of eye movement analysis for both the RAN conditions and the TOWRE was a symbol on a grid: a digit, a word, or an object. Five participants from the original sample were excluded due to technical failures or participants’ unstable performance during the test. The exclusion criteria were: (a) either the participant looked out of too many areas of interest and/or (b) the participant did not keep their head still during reading. A total of 37 participants were included in the final analyses. All the analyses performed were run on these 37 samples (*n* = 37). The final analyses in the study included repeated measures ANOVA, correlation analyses, and regression analyses (performed in SPSS). Table 3 illustrates the descriptive statistics for all the variables in the study across RAN-related tasks and the word reading task (TOWRE).

To examine the effect of animacy (or digit type) and response type on participants’ fixation duration and the interaction effects between categories of stimuli and response type, we ran separate two-way repeated measures ANOVAs on the digit and object tasks (see Table 4).

As shown in Table 5 and Table 6, for object RAN, the results of the two-way repeated measures ANOVA indicated that there is a significant main effect of animacy on participants’ fixation durations (F (1,36) = 6.65, *p* = 0.014, *η*_p_^2^ = 0.156). There is also a considerable effect of response type (naming versus categorizing) on fixation times (F (1,36) = 61.39, *p* < 0.01, *η*_p_^2^ = 0.630). Most importantly, the results confirmed a significant interaction effect between animacy and response type (F (1,36) = 12.26, *p* = 0.001, *η*_p_^2^ = 0.254). For digit-RAN, we observed a similar pattern: there is a significant effect of digit type on participants’ fixation latencies (F (1,36) = 13.08, *p* < 0.001); for response type on fixation durations (F (1,36) = 74.66, *p* < 0.001); and for the interaction effect between digit type and response type (F (1,36) = 7.30, *p* = 0.010), with respective partial eta-squared values of 0.266, 0.675, and 0.169, respectively.

Table 7 reports all Pearson correlation coefficients between eye movement variables in the RAN conditions and those in TOWRE. These analyses revealed significant correlations between eye movement variables in the RAN-related tasks and in the reading task: longer reading times, greater fixation latencies, and shorter time for making saccades in TOWRE were associated with longer naming times, greater fixation latencies, and shorter times for making saccades in the RAN-related tasks. These correlations remained significant across all the RAN tasks, including the categorization tasks where participants did not need to name aloud the visual stimuli.

We also calculated correlations between eye movement variables and naming times within the same RAN tasks. Longer naming times in RAN were correlated with longer fixation durations and smaller total saccade duration (Table 8).

Table 9, Table 10 and Table 11 show correlation coefficients of different types of eye movement variables among the four types of RAN conditions. Overall, eye movement variables were consistent across tasks at the individual level.

In order to examine the impact of four different versions of RAN tasks on TOWRE reading speed, we performed four sets of hierarchical multiple regressions (Models 1–4 in Table 12). With hierarchical multiple regressions, we need to study the possible presence of multicollinearity by means of a variance inflation factors (VIF) (test and tolerance analysis for total fixation duration and eye movement variables across four conditions of RAN as we observe a number of bivariate correlations between the variables (Table 4, Table 5, Table 6, Table 7, Table 8 and Table 9). However, the VIF test and tolerance analysis have indicated that multicollinearity was not a concern for our regression analyses (RAN Object Naming, Tolerance = 0.840, VIF = 1.190; RAN Object Categorization, Tolerance = 0.833, VIF = 1.200; RAN Digit Naming, Tolerance = 0.705, VIF = 1.419; RAN Digit Categorization, Tolerance = 0.956, VIF = 1.045). In Model 1 and Model 3, we examined the effects of eye movement variables in conventional serial RANs (i.e., object grids and digit grids) on word reading speed. In Model 2 and Model 4, we examined the effects of eye movement variables in the modified serial RANs (i.e., categorization tasks using the same object and digit grids in Model 1 and Model 3) on word reading speed. Results from the regression analyses revealed that a combination of fixation duration and eye movements from the conventional RAN conditions was a good predictor of word reading speed, accounting for nearly 36.2% and 35.2% of the variance, respectively. In categorization tasks where there was no demand for phonological retrieval, eye movement variables in RAN still explained 33.8% and 41.7% in RAN object categorization and RAN digit categorization, respectively.

## 4. Discussion

In this study, we examined the impact of semantic processing and eye movement variables in rapid naming and reading tasks. We hypothesized that semantic activation is an essential component, even in conventional rapid naming tasks where participants are simply required to name aloud the visual stimuli presented. Thus, rapid and accurate performance in conventional rapid naming tasks should be the combination of rapid and efficient retrieval of both phonological units and semantic properties of the stimuli from the mental lexicon. In order to test this, we used RAN grids that consisted of visual stimuli including an equal number of living and non-living objects. We used this design to test the effects of animacy, response types on participants’ fixation durations, and interaction effects between animacy and response type. 

In our results, we observed a significant impact of animacy and response type on fixation durations in all conditions, even in conditions where only phonological retrieval should be stipulated. We can conclude that semantic processing may be an essential component of conventional rapid naming tasks. This corresponds to the view that semantic integration and activation of the stimuli also play a role in defining the relationship between RAN and reading [8]. In this respect, RAN and reading measures are correlated, because both require rapid and accurate retrieval of phonological representations and semantic properties of visual stimuli. As a result, RAN is a robust predictor of reading abilities in both word decoding and text reading. Text reading requires several additional processes, apart from word recognition, such as syntactic processing, discourse analysis, inferential logic, world knowledge, semantic skills, and working memory [31]. Therefore, studies that use sentence or paragraph reading tend to report equal amounts of variance in reading explained by RAN than those that use word reading tasks. 

At the theoretical level, the mental distinction between animate and inanimate objects has been well documented since early infancy [39]. Recognition and activation of semantic properties are argued to be exclusive to human cognitive processes. According to the well-known spreading activation theory of Collins and Loftus (1975), different types of semantic features of the stimuli exert different effects on word recognition [40]. In this sense, should rapid naming tasks also activate semantic processing, we expect a considerable difference in fixation latencies between animate stimuli and inanimate stimuli, since animacy plays a significant role in defining semantic properties of words and stimuli [41]. The two-way repeated measures ANOVA confirmed the semantic effects on fixation durations in both conventional naming and in categorizing tasks. 

Another aim in the current study was to explore the extent to which eye movements contribute to the relations between RAN and reading. Empirical studies in the literature have found that RAN-related tasks tap intonon-phonological and more general cognitive processes, such as executive functioning, and parafoveal coordination. According to the visual scanning hypothesis, RAN accounts for a large proportion of variance in reading, since eye movement patterns in rapid naming are similar to those registered during reading. RAN and reading are related because they both involve the ability to coordinate eye movements across different stimuli on a printed page, ideally in languages where reading direction is left-to-right We expected that longer fixation durations and shorter eye movement durations will also be linked to longer fixation durations and shorted eye movement durations in reading tasks. Many previously mentioned studies have used sentence and paragraph reading as a measure to investigate oculomotor control. We used word reading tasks primarily for two reasons: (1) word reading is a purer measure of decoding words and therefore should exhibit stronger effects with RAN. Sentence and paragraph reading involve not only word decoding, but also language comprehension, inferences, and world knowledge; (2) In the word efficiency reading task (i.e., TOWRE), a participant has to read words in columns and in a top-to-bottom direction instead of left-to-right. It is therefore interesting to see whether eye movements between RAN and reading are still similar in this case. If this is true, the visual scanning hypothesis should be revised: the coordination of eye movements establishes the relation between RAN and reading, and this coordination is independent of direction. 

On the flip side, we also found that eye movement patterns in RAN and reading are similar. Longer fixations and shorter saccades in RAN are associated with longer fixations and shorter saccades in a word reading task. As previously mentioned in the literature, eye movement variables were found to be highly correlated with those in sentence reading tasks [24], in paragraph reading tasks [24,34], and now in the current word reading task, and even in categorization tasks that do not require explicit articulation of symbol names. Some studies have found that eye movement patterns between rapid naming and reading remain similar when participants are asked to read backward from right to left [42]. In addition, the results presented here suggested that eye movement patterns between the word reading and RAN tasks remain strongly correlated, even when the participants were asked to read words from top to bottom. Taken together, these results call for modifications to the visual scanning hypothesis. As long as participants have to name symbols in a serial manner, eye movements between RAN and reading are still strongly related, regardless of whether they are left-to-right, right-to-left, or top-to-bottom directions. 

Like previous studies in the literature, our study still has some limitations that might have affected the results. Because of time pressure and insufficient resources, we did not record the number of words that were read correctly and incorrectly in the word reading test (TOWRE). At the time of data collection, we could not get a version of the word reading test that was compatible with the resolution and the screen size of the Tobii eye- tracker. We were initially interested in fluency, speed, and eye movement variables, rather than accuracy. Thus, we did not examine if the subsets of colors and objects lose its predictive value of reading accuracy in adults, and whether the RAN categorization tasks are still related to the participants’ reading score in the TOWRE. At the beginning of our experiment, we predicted that the categorization tasks may not predict participants’ accuracy scores well. An array of studies in the RAN-reading literature found that articulation alone does not account for variance in reading abilities. For example, Georgiou et al. (2013) found that RAN Cancellation and RAN Yes/No did not predict reading abilities in their participants [25]. Therefore, we conclude that oral production of the names of the symbols on RAN grids is exclusive to the RAN-reading relationship, and that articulation alone does not explain that strong relation. 

Due to limited empirical research on semantic processing in RAN and semantic deficits in dyslexia, it is difficult to explain and contrast our findings using existing literature. However, our study yields at least one important implication for the role of semantic processing in dyslexia. According to the Lexical Quality Hypothesis, semantics is among the vital components that contribute to the efficient and successful retrieval of words from the mental lexicon [43]. Previous studies recorded semantic deficits in dyslexic readers because of their impaired performance on modified rapid naming tasks (e.g., saying yes/no to animate/inanimate objects), suggesting that this type of deficit may be unique to dyslexia [44]. In a similar vein, our results suggestthat semantic deficits in dyslexia may actually be dependent on phonological processing deficits. One possible explanation for semantic processing problems in dyslexic readers may lie in the association of form (phonology and orthography) and meaning (which is integrated and consolidated knowledge in adult dyslexic readers). Problems with accessing the phonological representations of words alone may lead to breakdown within the bundle as a whole. Our findings are thus consistent with the Lexical Quality Hypothesis by Perfetti (2007) and the results reported in Rjthoven et al., (2018) [43,44]. Still, this explanation may be valid for alphabetic languages, where there is a close association between orthographic information and phonological representations of words. In non-alphabetic languages, such as Chinese or Japanese Kanji, there is a stronger link between orthography (e.g., strokes or characters) and semantics (e.g., each character represents a certain meaning); phonology is learnt by rote, rather than with a systematic mapping. Semantic processing deficits warrant further investigation of such languages.

## 5. Conclusions

The role of semantic integration and activation has not been systematically investigated in performance on RAN tasks in typical populations. The current study aimed to fill this gap. We examined how semantic and conceptual information is activated and integrated with other sources of information in on-line processing in both conventional rapid naming tasks of objects and digits, and in categorization tasks using the same stimuli. By doing so, we were also able to re-assess the extent to which eye movements accounted for the shared variance in RAN and reading tasks. Our results revealed significant impact of semantic distinctions on fixation durations in all conditions, even in conditions where only phonological retrieval should be stipulated. We can conclude that semantic processing may be an essential component of conventional rapid naming tasks. This is consistent with the view that semantic integration and activation of the stimuli play a role in defining the relationship between RAN and reading. These results also have practical applications for the way RAN tasks are administered and the way participant performance is interpreted.

## Figures and Tables

**Table 1:** Psycholinguistic semantic properties of 6 stimuli in the study (This information is available at the English Project Lexicon website: https://elexicon.wustl.edu/, accessed on 28 June 2021).

	Concreteness	Semantic Neighborhood Density	Semantic Neighbors	Semantic Diversity	Age of Acquisition	Body-Object Interaction	Emotional Valence	Emotional Arousal
chicken	4.800	0.592	2861	1.508	3.260	5.500	6.170	3.200
fish	5.000	0.651	6757	1.395	4.050	5.593	6.420	3.330
frog	5.000	0.568	676	1.576	4.320	5.125	5.840	4.070
key	4.890	0.689	9126	2.100	3.580	6.280	6.220	3.900
pencil	4.880	0.530	0	1.565	4.060	5.870	5.650	3.110
star	4.690	0.681	8.613	1.618	3.890	1.917	7.470	5.500

**Table 2:** Variables in the study, their interpretation and method of measurement (all in seconds).

Name of the Variable	Interpretation of the Variable	Method of Measurement
Total Naming Time	The total amount of time that a participant needed to name aloud all the visual stimuli they saw on a grid in a trial	We measured this variable by subtracting the end time at which a participant finished naming the last stimulus in the grid with the onset time at which a participant was shown the first stimulus
Total Fixation Duration	The total amount of time that a participant fixated on all the visual stimuli on a grid	Total Fixation Duration is the summed duration of all fixations landing on the targets in a trial
Time engaged in Saccades	The total amount of time that a participant spent on making saccades and coordinating their eyes across the stimuli in a trial	We calculated Time engaged in eye movements by subtracting Total Fixation Duration from the Total Naming Time
Total Fixation Duration for Animate Objects	The total amount of time that a participant fixated on animate targets in a trial	Total Fixation Duration for Animate Objects is the summed duration of all fixations landing on targets that were classified as “animate” in a trial
Total Fixation Duration for Inanimate Objects	The total amount of time that a participant fixated on inanimate targets in a trial	Total Fixation Duration for Inanimate Objects is the summed duration of all fixations landing on targets that were classified as “inanimate” in a trial
Total Fixation Duration for Odd Digits	The total amount of time that a participant fixated on odd digits in a trial	Total Fixation Duration for Odd Digits is the summed duration of all fixations landing on digits that were classified as “odd” in a trial
Total Fixation Duration for Even Digits	The total amount of time that a participant fixated on even digits in a trial	Total Fixation Duration for Even Digits is the summed duration of all fixations landing on digits that were classified as “even” in a trial

**Table 3:** Descriptive statistics for all the variables in the study (*n* = 37) (in seconds).

Condition	Measure	Min	Max	Mean	SD
TOWRE (Word Reading)	Total Naming Time	19.04	47.27	27.29	5.70
	Total Fixation Duration	9.67	33.81	19.45	5.19
	Eye Movement Time	3.46	17.77	7.84	3.30
RAN-Object Naming	Total Naming Time	16.83	42.75	24.85	4.58
	Total Fixation Duration	10.50	29.00	18.31	4.60
	Eye Movement Time	2.82	18.90	6.54	3.61
RAN-Digit Naming	Total Naming Time	9.95	20.70	14.03	2.27
	Total Fixation Duration	3.46	14.82	9.59	2.40
	Eye Movement Time	1.64	13.58	4.44	2.36
RAN-Object Categorization	Total Naming Time	13.53	26.72	19.25	3.11
	Total Fixation Duration	8.77	19.63	14.10	3.15
	Eye Movement Time	2.44	12.81	5.14	2.48
RAN-Digit Categorization	Total Naming Time	15.00	35.25	21.96	4.40
	Total Fixation Duration	9.65	24.95	15.52	4.07
	Eye Movement Time	2.35	12.69	6.44	2.74

**Table 4:** Descriptive statistics from two-way repeated measures ANOVA for total fixation duration (measured in seconds) across animacy/digit type and response type.

Task	Animate		Inanimate	
	Mean	SD	Mean	SD
RAN-Object Naming	9.23	2.45	9.08	2.35
RAN-Object Categorization	6.58	1.79	7.52	1.61
	**Even**		**Odd**	
	**Mean**	**SD**	**Mean**	**SD**
RAN-Digit Naming	4.76	1.36	4.83	1.18
RAN-Digit Categorization	7.33	2.10	8.19	2.21

**Table 5:** Tests of Within-Subjects Contrasts from two-way repeated ANOVA for RAN Object.

Source	Animacy	Response	*df*	F	Sig.	Partial Eta Squared
Animacy	Linear		1	6.64	0.14	0.156
Error (Animacy)	Linear		36			
Response		Linear	1	61.38	<0.001	0.630
Error (Response)		Linear	36			
Animacy × Response	Linear	Linear	1	12.25	0.001	0.254
Error (Animacy × Response)	Linear	Linear	36			

**Table 6:** Tests of Within-Subjects Contrasts from two-way repeated ANOVA for RAN Digit.

Source	Digit	Response	*df*	F	Sig.	Partial Eta Squared
Digit	Linear		1	13.08	<0.001	0.266
Error (Digit)	Linear		36			
Response		Linear	1	74.66	<0.001	0.675
Error (Response)		Linear	36			
Digit × Response	Linear	Linear	1	7.299	0.010	0.169
Error (Digit × Response)	Linear	Linear	36			

**Table 7:** Pearson’s correlation between naming times and eye movement variables in RAN-related tasks and naming times and eye movement variables in word reading task (TOWRE).

Condition	Measure	Reading Time in TOWRE	Fixation Duration in TOWRE	Eye Movement Time in TOWRE
RAN-Object Naming	Naming Time	0.59 **	0.50 **	0.21
	Fixation Duration	0.30	0.57 **	−0.37 *
	Eye Movement Time	0.36 *	−0.08	0.74 **
RAN-Object Categorization	Naming Time	0.60 **	0.56 **	0.16
	Fixation Duration	0.49 **	0.62 **	−0.14
	Eye Movement Time	0.14	−0.08	0.38 *
RAN-Digit Naming	Naming Time	0.54 **	0.32 **	0.41 *
	Fixation Duration	0.04	0.24	−0.31
	Eye Movement Time	0.47 **	0.06	0.71 **
RAN-Digit Categorization	Naming Time	0.64 **	0.68 **	0.03
	Fixation Duration	0.46 **	0.62 **	−0.17
	Eye Movement Time	0.34 *	0.17	0.32

Note: * *p* < 0.05; ** *p* < 0.01.

**Table 8:** Pearson’s correlation between naming times and eye movement variables within the same RAN-related tasks.

Condition	Measure	Naming Time	Fixation Duration	Eye Movement Time
RAN-Object Naming	Naming Time	-	0.69 **	0.38 *
	Fixation Duration		-	−0.40 *
	Eye Movement Time			-
RAN-Object Categorization	Naming Time	-	0.68 **	0.38 *
	Fixation Duration		-	−0.40 *
	Eye Movement Time			-
RAN-Digit Naming	Naming Time	-	0.49 **	0.46 **
	Fixation Duration		-	−0.54 *
	Eye Movement Time			-
RAN-Digit Categorization	Naming Time	-	0.79 **	0.42 **
	Fixation Duration		-	−0.21
	Eye Movement Time			-

Note: * *p* < 0.05; ** *p* < 0.01.

**Table 9:** Pearson’s correlations of total naming times recorded across all the RAN-related tasks.

	RAN Object N	RAN Object C	RAN Digit N	RAN Digit C
RAN-ON	-	0.576 **	0.468 **	0.476 **
RAN-OC		-	0.256	0.657 **
RAN-DN			-	0.272
RAN-DC				-

Note: ** *p* < 0.01.

**Table 10:** Pearson’s correlations of fixation durations recorded across all the RAN-related tasks.

	RAN Object N	RAN Object C	RAN Digit N	RAN Digit C
RAN-ON	-	0.704 **	0.484 **	0.496 **
RAN-OC		-	0.441 **	0.737 **
RAN-DN			-	0.249
RAN-DC				-

Note: ** *p* < 0.01.

**Table 11:** Pearson’s correlations of eye movement time recorded across all the RAN-related tasks.

	RAN Object N	RAN Object C	RAN Digit N	RAN Digit C
RAN-ON	-	0.596 **	0.579 **	0.432 **
RAN-OC		-	0.424 **	0.477 **
RAN-DN			-	0.282
RAN-DC				-

Note: ** *p* < 0.01.

**Table 12:** Multiple hierarchical regressions for selected eye movement variables in Rapid Automatized Naming and total reading time in TOWRE.

Predictors Added	*R* ^2^	*R*^2^ Change	B	Beta	Sig. *F* Change	Sig.
**Model 1: Object Naming**						
Step 1						
Fixation Duration	0.092		0.38	0.30	0.069	0.69
Step 2						
Fixation Duration			0.657	0.530		0.001
Eye Movement			0.895	0.567		0.001
Fixation Duration + Eye Movement	0.362	0.270			0.000	0.000
**Model 2: Object Categorization**						
Step 1						
Fixation Duration	0.235		0.878	0.455	0.002	0.002
Step 2						
Fixation Duration			1.182	0.652		0.000
Eye Movement			0.943	0.410		0.009
Fixation Duration + Eye Movement	0.375	0.140			0.009	0.000
**Model 3: Digit Naming**						
Step 1						
Fixation Duration	0.002		0.101	0.042	0.803	0.803
Step 2						
Fixation Duration			1.012	0.426		0.014
Eye Movement			1.709	0.705		0.000
Fixation Duration + Eye Movement	0.352	0.350			0.000	0.001
**Model 4: Digit Categorization**						
Step 1						
Fixation Duration	0.214		0.649	0.463	0.004	0.004
Step 2						
Fixation Duration			0.785	0.560		0.000
Eye Movement			0.959	0.460		0.002
Fixation Duration + Eye Movement	0.417	0.202			0.002	0.000

**Figure A1:**
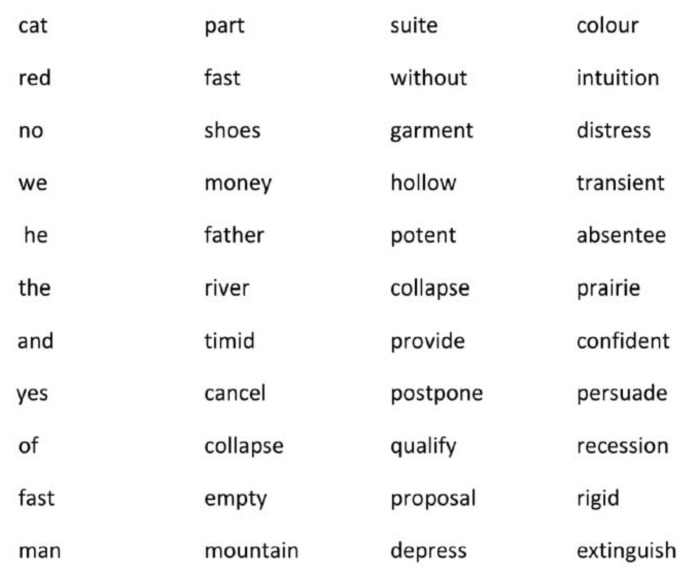
Test of Word Reading Efficiency (TOWRE).

**Figure A2:**
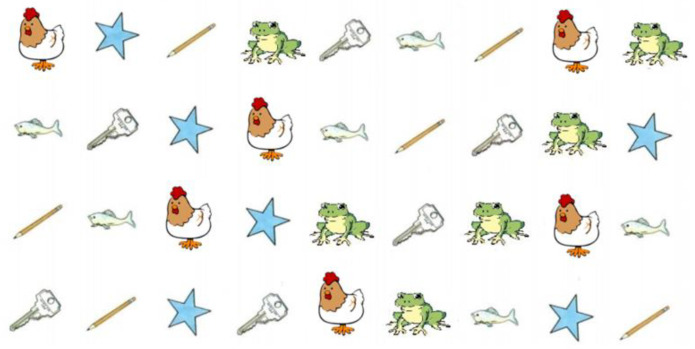
Serial Object Rapid Automatized Naming.

**Figure A3:**
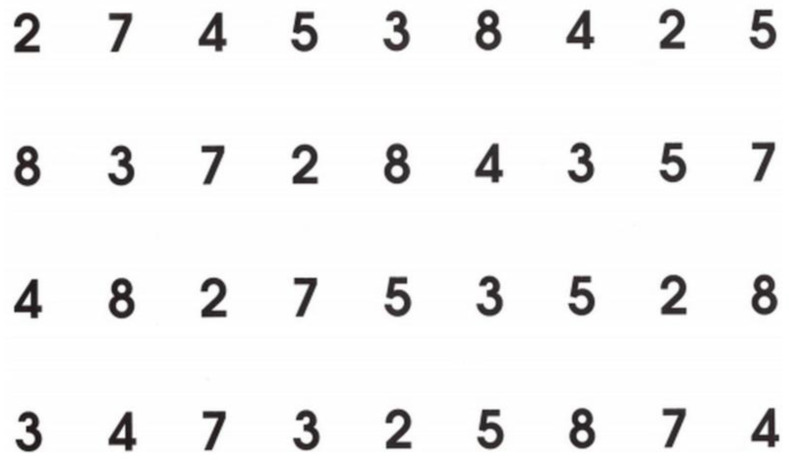
Serial Digit Rapid Automatized Naming.

## Data Availability

The data presented in this study are available on request from the corresponding author. The data are not publicly available due to GDPR constraints.
